# Comparison of perioperative outcomes between pure laparoscopic surgery and open right hepatectomy in living donor hepatectomy: Propensity score matching analysis

**DOI:** 10.1038/s41598-020-62289-0

**Published:** 2020-03-24

**Authors:** Ji Seon Jeong, Wongook Wi, Yoon Joo Chung, Jong Man Kim, Gyu-Seong Choi, Choon Hyuck David Kwon, Sangbin Han, Mi Sook Gwak, Gaab Soo Kim, Justin Sangwook Ko

**Affiliations:** 1 Department of Anesthesiology and Pain Medicine, Samsung Medical Center, Sungkyunkwan University School of Medicine, Seoul, Korea; 2 Surgery, Samsung Medical Center, Sungkyunkwan University School of Medicine, Seoul, Korea; 3 Department of General Surgery, Digestive Disease & Surgery Institute, Cleveland Clinic, Cleveland, Ohio USA

**Keywords:** Liver cirrhosis, Outcomes research

## Abstract

Pure laparoscopic donor right hepatectomy (PLDRH) is not a standard procedure for living donor liver transplantation but is safe and reproducible in the hands of experienced surgeons. However, the perioperative outcomes of PLDRH have not been fully evaluated yet. We used propensity score matching to compare the perioperative complications and postoperative short-term outcomes of donors undergoing PLDRH and open donor right hepatectomy (ODRH). A total of 325 consecutive donors who underwent elective, adult-to-adult right hepatectomy were initially screened. After propensity score matching, all patients were divided into two groups: PLDRH (n = 123) and ODRH (n = 123) groups. Perioperative complications and postoperative outcomes were compared between the two groups. Postoperative pulmonary complications were significantly more common in the ODRH than in the PLDRH group (54.5 vs. 31.7%, *P* < 0.001). The biliary complications (leak and stricture) were higher in PLDRH group than in the ODRH group (8% vs. 3%), but it failed to reach statistical significance (*P* = 0.167). Overall, surgical complication rates were similar between the two groups (*P* = 0.730). The opioid requirement during the first 7 postoperative days was higher in the ODRH group (686 vs. 568 mg, *P* < 0.001). The hospital stay and time to the first meal were shorter in the PLDRH than in the ODRH group (*P* = 0.003 and *P* < 0.001, respectively). PLDRH reduced the incidence of postoperative pulmonary complications and afforded better short-term postoperative outcomes compared to ODRH. However, surgical complication rates were similar in both groups.

## Introduction

Laparoscopic liver surgery has become widely accepted, affording many benefits including fewer overall complications, less blood loss, lower pain scores, better donor quality-of-life during the early postoperative period, and a shorter hospital stay, compared to open surgery^1–3^. As surgical techniques advanced over time, laparoscopic surgery for living donor liver transplantation (LDLT) became possible^2^. After the first successful reports of laparoscopic left lateral sectionectomy during adult-to-child LDLT in 2002^4^, many centers adopted the laparoscopic approach as the standard for living liver donors undergoing small graft resections^5,6^. However, the use of laparoscopic procedures to perform larger graft resections (such as right hepatectomies) was initially limited by technical complexities and the steep learning curve associated with the procedures. However, a series of laparoscopic right hepatectomies performed by experienced surgeons at living donor centers showed that the results were equivalent to those of open donor surgery^7^. Over time, the initial clinical acceptance of hand-assisted laparoscopic, donor right hepatectomy advanced to the adoption of pure laparoscopic donor right hepatectomy (PLDRH)^1,8^. Several preliminary studies suggested that PLDRH was safe and reproducible in the hands of experienced surgeons^7,9–11^. However, the available data focus principally on the incidence and nature of surgery-related complications^12^; neither perioperative complications nor postoperative short-term outcomes of PLDRH have been fully evaluated. Therefore, we used retrospective propensity score matching in this study to compare perioperative complications and postoperative short-term outcomes between PLDRH and open donor right hepatectomy (ODRH) patients.

## Methods

### Patients and study design

The Institutional Review Board of Samsung Medical Center approved this retrospective study (approval no. SMC 2018-07-068-001) and waived the requirement for written informed consent. All procedures in this study were performed in accordance with the relevant guidelines and regulations.

**Figure 1:**
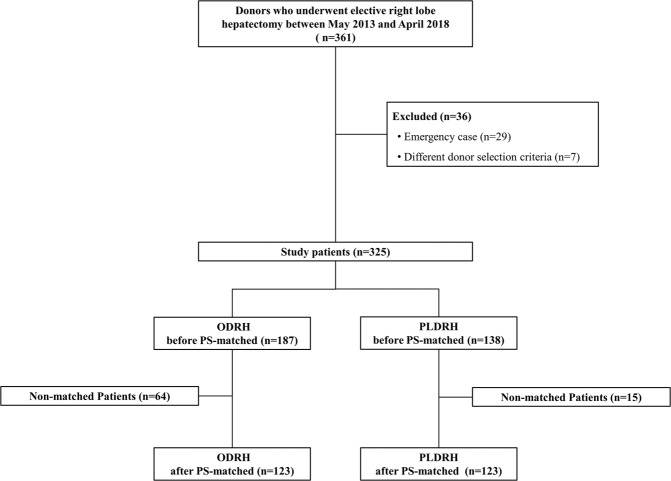
Flow diagram of the study. ODRH, open donor right hepatectomy; PLDRH, pure laparoscopic donor right hepatectomy; PSM, propensity score matching.

All data were collected from digitalized patient charts and the clinical data warehouse of Samsung Medical Center. Our institute commenced an LDLT program in 1996 and PLDRH was initiated in May 2013. Between May 2013 and April 2018, a total of 361 consecutive patients who underwent donor right hepatectomy or extended right hepatectomy during adult-to-adult LDLT were initially screened. Emergency cases (n = 29) and cases in which the donor selection criteria were unusual (n = 7) were excluded. The remaining 325 patients were divided into two groups by surgery type: ODRH (n = 187) and PLDRH (n = 138) groups. To overcome bias caused by differences in covariate distributions, we used one-to-one propensity score matching to ensure that the two groups were comparable. Finally, 123 patients who underwent ODRH and 123 who underwent PLDRH were enrolled. An overview of the study design is shown in Fig. 1.

### Donor selection

Our institutional selection criteria for ODRH when performing adult-to-adult LDLT are as follows: (1) Donor less than 65 years of age; (2) less than 30% macrosteatosis evident on frozen biopsy; and (3) expected remnant liver volume greater than 30%. Early on, PLDRH was performed only when the donor was younger than 60, and only when the donor elected to do so. In the first seven consecutive patients, only donors with normal vascular or biliary structures underwent PLDRH. Surgical techniques, benefits, and complications of both open and laparoscopic procedures were thoroughly explained to the donors before they made the choice. After these patients exhibited no major complications, we decided to expand the selection criteria. Currently, our donor selection criteria for ODRH and PLDRH do not differ, and PLDRH is now recommended to donors as the primary surgical technique. Therefore, we excluded the initial seven patients from analysis.

### Surgical technique

#### ODRH

Laparotomy was performed via incisions of both subcostal regions, combined with cephalic extensions. After dissecting ligaments around the liver, the right hepatic artery and portal vein were briefly clamped to define a demarcation line prior to parenchymal sectioning. An ultrasound device was used to detect the geographical relationship between the middle hepatic vein and the liver parenchyma. During parenchymal dissection, the middle hepatic veins of segments V and VIII were gently divided and ligated, while leaving sufficient length for reconstruction. After the hepatic vein trunk was encountered, parenchymal dissection was completed posteriorly. The hepatic artery, portal vein, and hepatic vein were divided, and the graft liver was delivered to a bench for perfusion. The remnant stumps of the hepatic and bile ducts were ligated or closed without narrowing.

#### PLDRH

A narrative overview on our PLDRH surgical technique was previously published by one of our surgeons^13^. Briefly, five trocar ports were placed as follows: one 12-mm port receiving a 30° optic device at the umbilicus; one 12-mm operative trocar each at the right midaxillary line and the midline; and two 5-mm trocars for instrumental assistance, one at the left midclavicular area and one in the subxiphoid region. Ultrasound observation followed by parenchymal sectioning was performed as described for ODRH. Parenchymal sectioning proceeded with the aid of an ultrasonic dissector (Sonicision^TM^; Medtronic, Minneapolis, MN, USA) and a cavitron ultrasonic surgical aspirator (CUSA® Excel; Integra LifeSciences, Plainsboro, NJ, USA). The major hepatic veins were saved for reconstruction. After completion of parenchymal dissection, the remnant bile duct stump was sutured. A Pfannenstiel incision of length 12–14 cm was created in the suprapubic area and the graft was retrieved in a plastic bag prior to perfusion.

### Anesthesia and monitoring

After initiation of standard anesthesia monitoring including electrocardiography, pulse oximetry, non-invasive blood pressure measurement, and monitoring of the bispectral index (BIS), 400 µg morphine was administered intrathecally through the level 4–5 lumbar intervertebral space^14^. Anesthesia was induced with 5 mg kg^−1^ sodium thiopental and 0.1 mg kg^−1^ vecuronium followed by tracheal intubation. Thereafter, the end-tidal carbon dioxide (ETCO_2_), oroesophageal temperature, and arterial blood pressure were monitored (the latter invasively, through a radial artery catheter). Dynamic indices that could be calculated using arterial blood pressure data (e.g., pulse pressure variation [PPV]) were evaluated throughout the operation. Anesthesia was maintained with inhaled isoflurane, with the BIS held between 40 and 60. Intravenous (IV) remifentanil was continuously infused (0.01–0.1 µg kg^−1^ min^−1^) to control blood pressure and heart rate. Hypotension (mean blood pressure decrease >20% from baseline) was treated with 5 mg ephedrine, and bradycardia (heart rate <50 beats min^−1^) was treated with 0.5 mg atropine. Neuromuscular blockade was monitored with the aid of a nerve stimulator attached to the adductor pollicis muscle. The thumb response to train-of-four (TOF) counts of 0 or 1 was monitored via continuous infusion of 0.8–1.0 µg kg^−1^ min^−1^ vecuronium. The mechanical ventilator was set to the volume-controlled ventilation mode with a tidal volume of 6–8 mL kg^−1^ and a positive end-expiratory pressure (PEEP) of 6 cm H_2_O, using a mixture of medical air and oxygen at a fresh gas flow rate of 2 L min^−1^ (fraction of inspired oxygen = 0.5). The ventilation frequency was adjusted to maintain the ETCO_2_ at 4.0–5.7 kPa. If the peak pressure exceeded 30 cm H_2_O, we changed the ventilator mode to pressure-controlled ventilation^15^. During PLDRH, the intraperitoneal pressure was adjusted to 12 ± 2 mmHg after inflation of the abdominal space, and the 30° reverse Trendelenburg position was established. Immediately after graft removal and stabilization of hemostasis, the CO_2_ gas supply was stopped and the patient was returned to the supine position.

A balanced crystalloid solution (Plasma solution A; CJ Healthcare, Seoul, Korea) was used as the intraoperative maintenance fluid. The fluid level was controlled to maintain normovolemia. A colloid solution comprising 6% (w/v) Volulyte (Fresenius Kabi, Bad Homburg, Germany) was infused if the attending anesthesiologist considered it necessary. Arterial blood gas analysis was performed every 2 h during the operation. Our institute guideline states that red blood cells should be transfused if hemoglobin levels fall below 8 g dL^−1^. However, no patient required an intraoperative transfusion. The body temperature was maintained at 36.5 °C by providing a warming blanket.

After completion of surgery, oropharyngeal suctioning was gently performed and 0.25 mg kg^−1^ pyridostigmine with 0.01 mg kg^−1^ glycopyrrolate were administered to reverse the neuromuscular blockade. After confirming that the TOF ratio was >0.9, respiration was adequate, and that the eyes opened in response to a verbal stimulus, the tracheal tube was removed.

### Pain control and discharge

For pain control, 400 µg of intrathecal morphine was administered prior to general anesthesia, because our prior study revealed that this afforded a superior analgesic effect^14^. Pethidine (25 mg via IV) was given prior to the end of surgery. After surgery, IV patient-controlled analgesia (PCA) was initiated in the post-anesthesia care unit (PACU). The PCA solution comprised 0.9% (w/v) normal saline with 15 µg mL^−1^ fentanyl, and was delivered at a rate of 1 mL h^−1^ with a 1-mL bolus dose and a 15-min lockout time. Patients with numeric pain rating scale (NPRS) scores of >3 despite the use of IV PCA received rescue IV analgesics. PACU anesthesiologists prescribed the type and dose of such analgesics. After PACU discharge, either or both oral and IV analgesics were prescribed, depending on the NPRS score, by the surgeon. To allow us to compare the opioid requirements of the two groups, all opioids prescribed during the first 7 postoperative days were converted to oral morphine equivalent doses (OMEDs). The donor was discharged at the discretion of the surgeon if conditions such as the oral feeding, normalization of laboratory results, removal of all drains, and postoperative pain control with oral analgesics were met.

### Outcomes

Our primary aim was to compare the perioperative complications and postoperative short-term outcomes of the two groups. Perioperative complications included intraoperative hypotension and bradycardia, postoperative nausea and vomiting (PONV) that require treatment with metoclopramide, pruritus that require treatment with pheniramine, postoperative pulmonary complications, and surgical complications. All patients underwent chest x-rays after surgery and complications were evaluated using European Perioperative Clinical Outcome definitions^16^. All postoperative complications were evaluated using the modified Clavien-Dindo classification^17^ and the Comprehensive Complication Index (CCI)^18^. Short-term postoperative outcomes were investigated during the hospital stay. Secondary outcomes were evaluated by monitoring intraoperative hemodynamic and respiration levels and surgical complications, and examining laboratory data obtained pre- and post-operatively.

### Statistical analyses

Continuous variables are presented as means ± standard deviations (SDs) or as medians with interquartile ranges (IQRs), as appropriate, and categorical variables are presented as percentages. The normality of continuous variables was assessed using the Shapiro-Wilk test, and parameters were compared between groups using the Student’s t-test or Mann-Whitney U-test, as appropriate. The chi-squared or Fisher’s exact test was employed to compare categorical variables.

To deal with bias attributable to the distinct distributions of covariates among patients in the two groups, we performed propensity score matching (one-to-one) using the nearest-neighbor method, with no replacement. The following covariates were analyzed: height, weight, age, sex, American Society of Anesthesiologists (ASA) physical status, graft type (right vs. extended right), surgeon, current smoker status, and any history of abdominal operation. The matching algorithm was based on logistic regression and tested by drawing histograms of the propensity scores and the standardized differences, and a dot plot of the standardized mean differences (Supplementary Fig. S1). Matching was considered appropriate when the difference in the logit of the propensity score between the nearest neighbors was within one caliper-width (0.1). All statistical analyses were performed using SPSS software (version 22; IBM Corporation, Armonk, NY, USA). The level of statistical significance (α) was set to 0.05.

## Results

We initially included 325 donors, 187 that underwent ODRH and 138 that underwent PLDRH. After propensity score matching, 123 donors remained in each group. The demographic and preoperative characteristics of patients before and after propensity score matching are summarized in Table 1. Open conversion was triggered by portal vein stenosis in two patients, portal vein injury in one, left bile duct injury in one, and inferior vena cava tearing in one.

**Table 1:** Demographic and preoperative characteristics of study patients before and after propensity score matching.

	Before propensity score matching				After propensity score matching			
	ODRH (n = 187)	PLDRH (n = 138)	*P-*value	SMD	ODRH (n = 123)	PLDRH (n = 123)	*P-*value	SMD
Age, years	31 (24–42)	30 (23–37)	0.170	−0.162	31 (23–39)	30 (23–42)	0.903	−0.016
Sex, n (%)			0.030	0.239			0.796	0.032
Male	120 (64.2)	72 (52.2)			73 (59.3)	71 (57.7)		
Female	67 (35.8)	66 (47.8)			50 (40.7)	52 (42.3)		
Height, cm	168.2 ± 8.5	167.0 ± 8.8	0.185	−0.147	168.2 ± 8.7	167.8 ± 8.9	0.725	−0.045
Weight, kg	66.5 ± 10.1	65.5 ± 10.6	0.379	−0.096	66.7 ± 10.5	65.8 ± 10.3	0.502	−0.085
ASA classification, n (%)			0.918	0.011			0.451	0.094
I	174 (93.0)	128 (92.8)			116 (94.3)	113 (91.9)		
II	13 (7.0)	10 (7.2)			7 (5.7)	10 (8.1)		
Surgeon, n (%)			0.195	−0.199			0.955	0.030
A	75 (40.1)	66 (47.8)			55 (44.7)	56 (45.5)		
B	25 (13.4)	11 (8.0)			10 (8.1)	11 (8.9)		
C	87 (46.5)	61 (44.2)			58 (47.2)	56 (45.5)		
Type of operation, n (%)			0.256	−0.110			0.734	0.035
Right hepatectomy	181 (96.8)	130 (94.2)			118 (95.9)	119 (96.7)		
Extended right hepatectomy	6 (3.2)	8 (5.8)			5 (4.1)	4 (3.3)		
Conversion to open approach	—	5 (3.6)			—	5 (4.1)		
Previous abdominal surgery, n (%)	32 (17.1)	24 (17.4)	0.948	−0.007	22 (17.9)	23 (18.7)	0.869	−0.021
Current smoker, n (%)	35 (18.7)	21 (15.2)	0.409	0.097	23 (18.7)	20 (16.3)	0.615	0.068

Data are presented as means ± standard deviations (SDs), medians with interquartile ranges (IQRs) in parentheses, or numbers with percentages in parentheses. ODRH, open donor right hepatectomy; PLDRH, pure laparoscopic donor right hepatectomy; BMI, body mass index; ASA, American Society of Anesthesiologists; SMD, Standardized mean difference.

### Intraoperative outcomes

Intraoperative outcomes are summarized in Table 2. Total operation time, anesthetic time, and time to graft procurement did not differ significantly between the two groups (*P* = 0.649, *P* = 0.165, and *P* = 0.206, respectively). The Pringle maneuver was performed on 92 of 123 (74.8%) patients in the ODRH group and 52 of 123 (42.3%) in the PLDRH group (difference between groups, 32.5%; 95% confidence interval [CI], 19.8–43.8%). This difference was statistically significant (*P* < 0.001). The estimated blood loss (EBL) was significantly more in the ORDH (*P* = 0.036), but amounts of intraoperative colloids infused were significantly more in the PLDRH (*P* = 0.017). No patient required an intraoperative transfusion in the both groups.

**Table 2:** Intraoperative characteristics of patients in ODRH and PLDRH groups.

	ODRH (n = 123)	PLDRH (n = 123)	*P*-value
Operation time, min	330 ± 68	335 ± 95	0.649
Anesthetic time, min	389 ± 70	404 ± 99	0.165
Total remifentanil dose, mg	1.12 ± 0.73	1.00 ± 0.80	0.271
Time to graft procurement, min	236 ± 56	246 ± 64	0.206
Pringle maneuver, n	92 (74.8)	52 (42.3)	<0.001
**Ventilation**			
Highest peak inspiratory pressure, cm H_2_O	17 (16–19)	24 (22–25)	<0.001
Highest plateau airway pressure, cm H_2_O	15 (14–17)	21 (19–22)	<0.001
Tidal volume, mL	478 ± 90	453 ± 85	0.027
Driving pressure, cm H_2_O	11 (10–13)	16 (15–17)	<0.001
Lung compliance, mL cm H_2_O^−1^	43 ± 9	30 ± 6	<0.001
Pressure-controlled ventilation, n	0 (0)	2 (1.9)	0.498
**Oxygenation**			
FiO_2_	0.55 ± 0.04	0.55 ± 0.05	0.835
PaO_2,_ mmHg	317 (293–334)	303 (254–326)	0.001
PaO_2_/FiO_2_ ratio	574 (538–612)	561 (485–594)	<0.001
**Fluid management**			
Crystalloids, mL	1988 ± 553	2058 ± 621	0.538
Colloids, mL	459 ± 137	504 ± 120	0.017
Maximum PPV, %	13 (10–16)	22 (17–26)	<0.001
Estimated blood loss, mL	334 ± 155	300 ± 160	0.036
Urine output, mL	310 (240–495)	330 (235–485)	0.931
Macrosteatosis >15%, n	4 (3.3)	4 (3.3)	1.000
Microsteatosis >15%, n	14 (11.4)	5 (4.1)	0.054
Wound PCA, n	11 (8.9)	15 (12.2)	0.346

Data are presented as means ± SDs, medians with IQRs in parentheses, or numbers with percentages in parentheses. FiO2, fraction of inspired oxygen; PaO2, partial pressure of arterial oxygen; PEEP, positive end-expiratory pressure; PPV, pulse pressure variation; PCA, patient-controlled analgesia.

**Figure 2:**
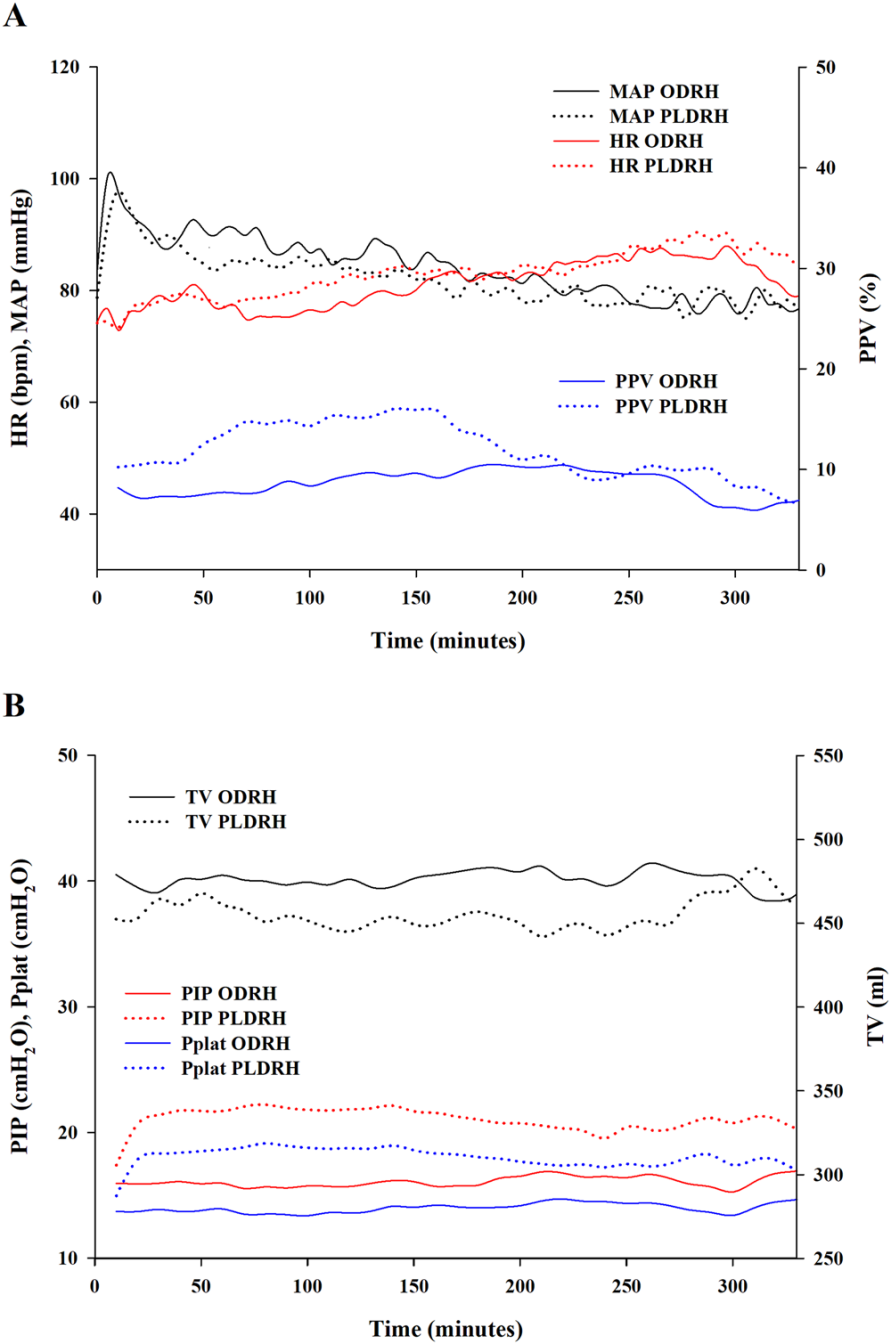
(**A**) The intraoperative hemodynamic parameters monitored in ODRH and PLDRH patients. (**B**) The intraoperative respiratory parameters monitored in ODRH and PLDRH patients. MAP, mean arterial pressure; HR, heart rate; PPV, pulse pressure variation; TV, tidal volume; PIP, peak inspiratory pressure; Pplat, plateau airway pressure.

The intraoperative respiratory and hemodynamic data are shown in Fig. 2. The median driving pressure (DP; the plateau pressure minus the PEEP) was 11 (10–13) cm H_2_O in the ODRH group and 16 (15–17) cm H_2_O in the PLDRH group. The median difference of 5 cm H_2_O had a 95% CI of 4–5 cm H_2_O and was significant (*P* < 0.001). Mean lung compliance (tidal volume/DP) values for the ODRH and PLDRH groups were 43 ± 9 and 30 ± 6 mL cm H_2_O^−1^, respectively. The mean difference of 14 mL cm H_2_O^−1^ had a 95% CI of 12–16 mL cm H_2_O^−1^ and was significant (*P* < 0.001). Two donors in the PLDRH group required pressure-controlled ventilation because they exhibited peak inspiratory pressure of more than 30 cm H_2_O. The median maximum PPVs of the ODRH and PLDRH groups were 13 (10–16)% and 22 (17–26)%, respectively. The median difference of 8% had a 95% CI of 7–10% and was significant (*P* < 0.001).

### Perioperative complications

All perioperative complications observed are summarized in Table 3. The frequencies of intraoperative hypotension and bradycardia, and postoperative PONV and pruritus, did not differ significantly between the two groups (*P* = 0.242, *P* = 0.151, *P* = 0.797, and *P* = 0.166, respectively). Postoperative pulmonary complications were significantly more common (*P* < 0.001) in the ODRH (54.5%) than in the PLDRH (31.7%) group (difference between groups, 22.8%; 95% CI, 9.8–34.7%). Specifically, atelectasis was significantly more prevalent (*P* = 0.009) in the ODRH (18.7%) than in the PLDRH (7.3%) group (difference between groups, 11.3%; 95% CI, 2.4–20.4%).

**Table 3:** Number of ODRH and PLDRH patients exhibiting perioperative complications.

	ODRH (n = 123)	PLDRH (n = 123)	*P-*value
Intraoperative hypotension	12 (9.8)	18 (14.6)	0.242
Intraoperative bradycardia	20 (16.3)	29 (23.6)	0.151
PONV^*^	69 (56.1)	71 (57.7)	0.797
Pruritus^†^	11 (8.9)	18 (14.6)	0.166
**Postoperative pulmonary complications**			
Pleural effusion	41 (33.3)	29 (23.6)	0.090
Atelectasis	23 (18.7)	9 (7.3)	0.009
Pneumonia	2 (1.6)	1 (0.8)	1.000
Overall	67 (54.5)	39 (31.7)	<0.001
**Surgical complications**			
Bile duct leakage	3 (2.4)	7 (5.7)	0.334
Bile duct stricture	1 (0.8)	3 (2.4)	0.622
Hepatic artery bleeding	0 (0)	1 (0.8)	1.000
Portal vein narrowing	0 (0)	1 (0.8)	1.000
Diaphragmatic hernia	1 (0.8)	0 (0)	1.000
Fluid collection	6 (4.9)	2 (1.6)	0.281
Wound complications	8 (6.5)	7 (5.7)	0.790
Overall	19 (15.4)	21 (17.1)	0.730

Data are presented as numbers with percentages in parentheses. PONV, postoperative nausea and vomiting.

^*^Patients required treatment with metoclopramide.

^†^Patients required treatment with pheniramine.

Surgical complications did not differ significantly between the two groups (difference between groups, 1.6%; 95% CI, −8.3–11.5%; *P* = 0.730). Biliary complications (leak and stricture) was 4 of 123(3.3%) in the ODRH group and 10 of 123(8.1%) in the PLDRH group (*P* = 0.167). One ODRH patient underwent re-operation to treat diaphragmatic hernia. Four PLDRH patients underwent re-operations, one to treat hepatic artery bleeding, two to treat bile duct leakage, and one to treat portal vein thrombosis.

### Postoperative short-term outcomes

The short-term postoperative outcomes are summarized in Table 4. Mean OMEDs over the first 7 postoperative days for the ODRH and PLDRH groups were 686 ± 253 and 568 ± 126 mg, respectively. The mean difference of 116 mg had a 95% CI of 66–167 mg and was significant (*P* < 0.001), but NPRS in the PACU score did not differ significantly between the two groups (*P* = 0.573). Patients in the ODRH group had a significantly longer median postoperative hospital stay (10 [8–12] days; *P* = 0.003), time to the first meal after surgery (3 [2,3] days; *P* < 0.001), and time to JP removal after surgery (6 [5–8] days; *P* = 0.005), compared to patients in the PLDRH group (9 [8–11] days, 1 [1,2] day, and 6 [4–8] days, respectively). The respective median difference was 1 day for each comparison, and the respective 95% CIs were 0–2, 1–2, and 0–1 days. However, re-admission rates did not differ between the two groups (*P* = 0.634).

**Table 4:** Postoperative short-term outcomes in ODRH and PLDRH patients.

	ODRH (n = 123)	PLDRH (n = 123)	*P-*value
Hospital stay (days)	10 (8–12)	9 (8–11)	0.003
Time to first meal (days)	3 (2–3)	1 (1–2)	<0.001
Time to JP removal (days)	6 (5–8)	6 (4–8)	0.031
NPRS in the PACU	6 (3–7)	5 (3–6)	0.573
OMED over the first 7 days (mg)	686 ± 253	568 ± 126	<0.001
Use of diuretics (no. of patients)	27 (22.0)	27 (22.0)	1.000
Re-admission (no. of patients)	11 (8.9)	8 (6.5)	0.634
CCI	10.5 ± 9.4	8.6 ± 10.1	0.155
Clavien-Dindo classification (no. of patients)			0.618
Grade I	13 (10.6)	13 (10.6)	1.000
Grade II	10 (8.1)	6 (4.9)	0.301
Grade IIIa	11 (8.9)	10 (8.1)	0.820
Grade IIIb	1 (0.8)	4 (3.3)	0.370

Data are presented as means ± SDs, medians with IQRs in parentheses, or numbers with percentages in parentheses. JP, Jackson-Pratt drain; NPRS, numerical pain rating scale; PACU, postoperative care unit; OMED, oral morphine equivalent dose; CCI, comprehensive complication index.

**Figure 3:**
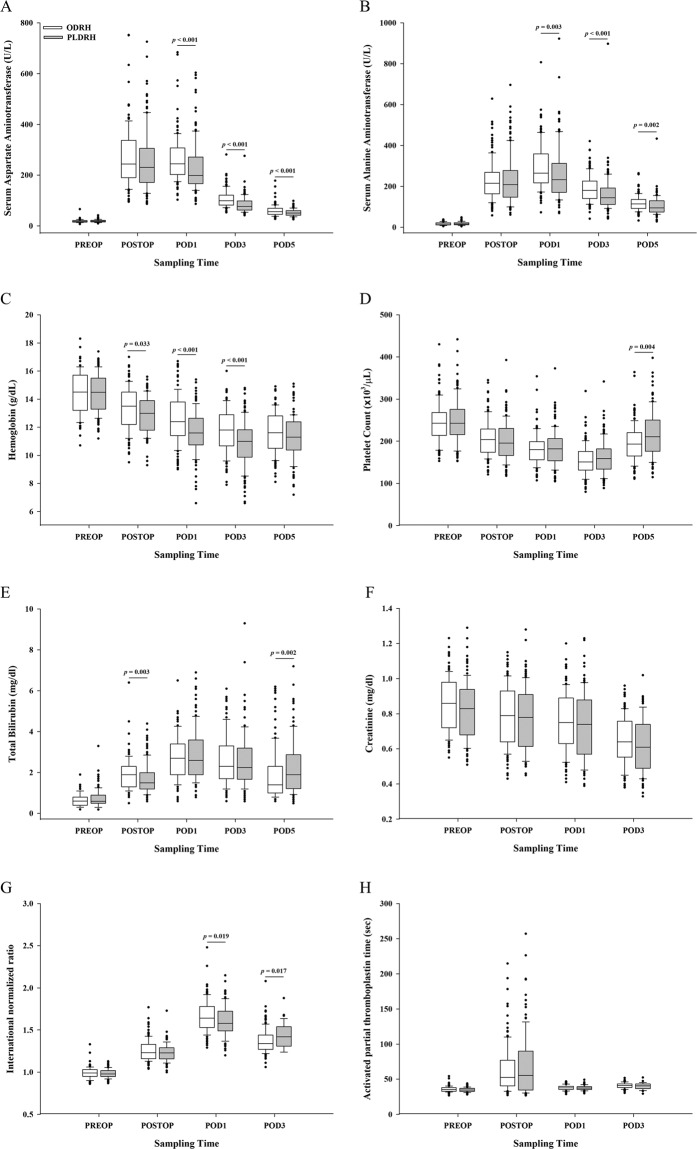
Laboratory findings during the pre- and postoperative periods. (**A**) Serum aspartate aminotransferase levels; (**B**) Serum alanine aminotransferase levels; (**C**) Hemoglobin levels; (**D**). Platelet counts; (**E**). Total bilirubin levels; (**F**). Creatinine levels; (**G**). International normalized ratios; (**H**). Activated partial thromboplastin times. PREOP, pre-operative period; POSTOP, immediate postoperative period; POD1, postoperative day 1; POD3, postoperative day 3; POD5, postoperative day 5.

Pre- and post-operative laboratory data are shown in Fig. 3. During postoperative days 1, 3, and 5, serum aspartate aminotransferase (Day 1, *P* < 0.001; Day 3, *P* < 0.001; Day 5, *P* < 0.001) and alanine aminotransferase (Day 1, *P* = 0.003; Day 3, *P* < 0.001; Day 5, *P* = 0.002) levels were lower in PLDRH patients. PLDRH patients exhibited lower hemoglobin levels on immediate postoperative period, postoperative days 1, and 3 (*P* = 0.033, *P* < 0.001, and *P* < 0.001, respectively).

## Discussion

In this retrospective study using propensity score matching, we compared the perioperative complications and postoperative short-term outcomes of PLDRH and ODRH patients. Living donors undergoing PLDRH exhibited better postoperative outcomes, including fewer postoperative pulmonary complications, less opioid consumption until postoperative day 7, and a shorter hospital stay. However, surgical complications as assessed using the Clavien-Dindo system did not differ significantly between the two groups.

Laparoscopic procedures employed for graft resections (such as right hepatectomy) continue to be highly demanding technically. Thus, their use remains both limited and controversial because of concerns about donor safety. Although hand-assisted, laparoscopic, right lobe donor hepatectomy through a subxyphoid vertical incision was introduced in 2006^19^, PLDRH has only recently been attempted in a few highly specialized centers^20^. Preliminary studies of these attempts revealed that the postoperative short-term outcomes were comparable to those of the open approach^7,9–11^. In particular, Kim *et al*. demonstrated the feasibility of pure laparoscopic procedures in three donors undergoing right hepatectomies^21^. Pure laparoscopic procedures has several advantages compared to conventional open surgery, including less wound morbidity and faster recovery^21^. More recently, Kwon *et al*. reported that, after careful donor selection, and using standardized surgical methods, pure laparoscopic donor hepatectomy for adult LDLT was safe and benefitted the donors^22^. Similarly, we found here (in the largest such study to date) that PLDRH donors had lower opioid requirements until postoperative day 7 than the ODRH donors. In addition, total hospital stay, time to first meal, and time to JP removal were shorter in the PLDRH than in the ODRH group.

Frequencies of perioperative complications including PONV and pruritus were similar between the two groups. However, the incidence of overall postoperative pulmonary complications was lower in donors undergoing PLRDH. Pulmonary complications are common after LDLT, particularly after right hepatectomy^23^. We recorded postoperative pulmonary complications in 106 patients (44%). During laparoscopic abdominal surgery, the increased abdominal pressure reduces the functional residual capacity and oxygenation extent more than during open surgery^24^. However, we found that the oxygenation and ventilation status of PLDRH and ODRH patients were similar. Also, despite the unfavorable intraoperative oxygenation and ventilation status of PLDRH patients, the incidence of postoperative pulmonary complications was only 60% of that of the ODRH group. Several explanations are possible. First, the reverse Trendelenburg position may offset the decrease in functional residual capacity that develops secondary to the pneumoperitoneum. Second, an intraoperative recruitment maneuver followed by PEEP adjustment effectively improves respiratory mechanics and oxygenation during laparoscopic surgery^25,26^. We performed recruitment maneuvers intermittently but routinely adjusted the PEEP continuously during laparoscopic surgery. Third, Jeong *et al*. found that open abdominal surgery was associated with more pulmonary complications than laparoscopic abdominal surgery^27^. It was suggested that this reflected less direct damage to the diaphragm and respiratory muscles during surgery, and less postoperative pain facilitating deep breathing and active coughing during recovery. Indeed, we found that the opioid consumption during the first 7 postoperative days was significantly lower in the PLDRH group, supporting the arguments made above. Therefore, healthy donor lung status, the use of intraoperative recruitment maneuvers, and better postoperative rehabilitation of the PLDRH group may have reduced postoperative pulmonary complications despite the poorer (compared to the ODRH group) intraoperative respiratory mechanics. However, only less than 10% of the pulmonary complications needed treatment.

As living liver donors are usually young and healthy, their cosmetic outcomes are important. DuBay *et al*. reported that, in younger donors, these outcomes and the perceived body image after adult, right lobe, living liver donation were significantly lower than those after renal donation; liver donors reported significantly poorer health-related quality-of-life^28^. PLDRH is most helpful in this context, because the small suprapubic incision created for graft extraction and the resultant scar can be covered by underwear or pants; this is not the case after open, hand-assisted laparoscopic hepatectomy.

The major postoperative complication (grade IIIb or higher) that required re-operation were more common in the PLDRH than in the ODRH group, but differences were not statistically significant. In particular, the incidence of postoperative biliary complications was two times higher in the PLDRH than in the ODRH group. Given the complexity and novelty of the former procedure, a higher incidence of major complications may seem inevitable. However, an experienced laparoscopic surgeon can reduce the incidence of such complications. Lee *et al*. reported a subgroup analysis of donors who underwent PLDRH; the major complication rate was 4.7% in the initial group but 0% in the most recent group^10^. Suh *et al*. also reported that modern technical developments such as three-dimensional laparoscopes and real-time, indocyanine green, near-infrared fluorescence cameras brought about substantial benefits^7^. These developments greatly aid laparoscopic surgeons. PLDRH can substitute for open surgery if it is performed by an experienced team using the best available surgical devices.

Our study had certain limitations. First, because this was a single-center retrospective work on a novel laparoscopic technique, there may have been selection bias. When we initially commenced PLDRH, the selection criteria differed between the two groups. Therefore, we excluded the initial seven cases. Also, we used propensity score matching to minimize the bias in our retrospective study; thus, facilitating more accurate between-group comparisons. Second, not all intraoperative events and complications may have been identified because we referred only to electronic medical records and assessed no long-term outcomes other than re-admission rate. Third, we compared donor outcomes only by surgery type. Thus, further studies on recipient graft outcomes by donor surgical technique are required.

In conclusion, the use of PLDRH instead of ODRH when performing adult-to-adult LDLT led to improved early postoperative outcomes and reduced postoperative pulmonary complications. The incidence of surgical complications was similar in both groups. Therefore, PLDRH is a viable alternative to ODRH when performed by experienced surgeons.

## Supplementary information

Supplementary Figure S1.

## Data Availability

The datasets generated during and/or analysed during the current study are available from the corresponding author on reasonable request.
