# Supplementary material for: Comparison of perioperative outcomes between pure laparoscopic surgery and open right hepatectomy in living donor hepatectomy: Propensity score matching analysis
Source: Sci Rep. 2020 Mar 24;10:5314. doi: 10.1038/s41598-020-62289-0 (PMC7093441; doi:10.1038/s41598-020-62289-0)

**Comparison of perioperative outcomes between pure laparoscopic surgery and open right hepatectomy in living donor hepatectomy: Propensity score matching analysis**

J. S. Jeong^1†^, W. Wi^1†^, Y. J. Chung^1^, J. M. Kim^2^, G. S. Choi^2^, C. H. D. Kwon^3^, S. Han^1^, M. S. Gwak^1^, G. S. Kim^1^, and J. S. Ko^1*^

*Department of* ^1^*Anesthesiology and Pain Medcine, and* ^2^*Surgery, Samsung Medical Center, Sungkyunkwan University School of Medicine, Seoul, Korea*

^3^*Department of General Surgery, Digestive Disease & Surgery Institute, Cleveland Clinic, Cleveland, Ohio, USA*

**Table of Contents for Supplementary Materials**

| Supplementary Figure S1 | 2 |
| --- | --- |

**Supplementary Figure S1.** A) Histogram of propensity scores and B) Dot plot of the propensity scores of patients with open and laparoscopic donor right hepatectomy showing individual units in the dataset and whether they were matched or discarded. C) Dot plot of standardized mean differences before and after propensity score matching. Treat and Treated units: patients with laparoscopic donor right hepatectomy; Control and Control units: patients with open donor right hepatectomy.

**
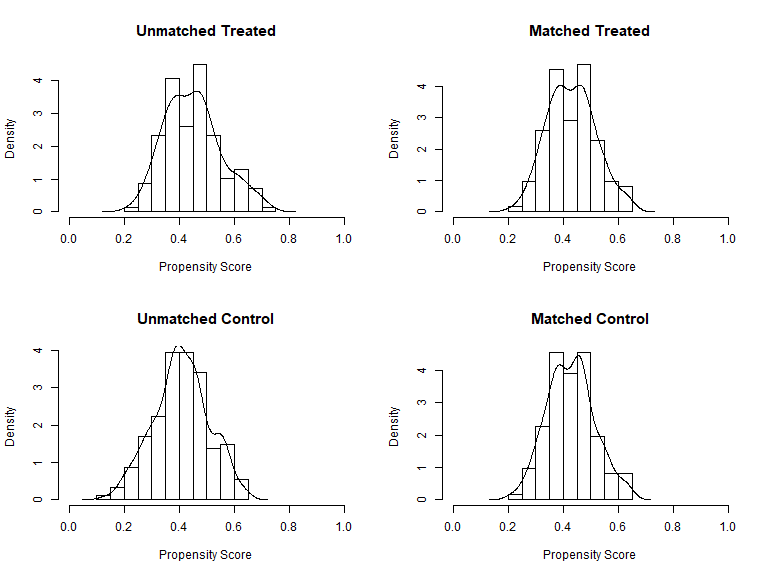
A)**

**B)**


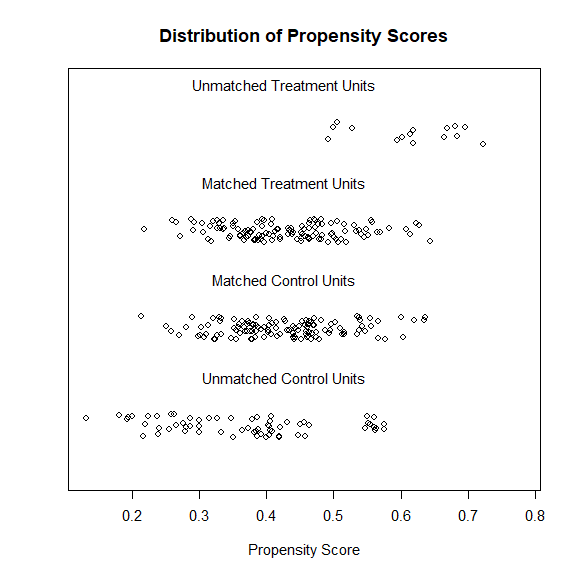


**C)**


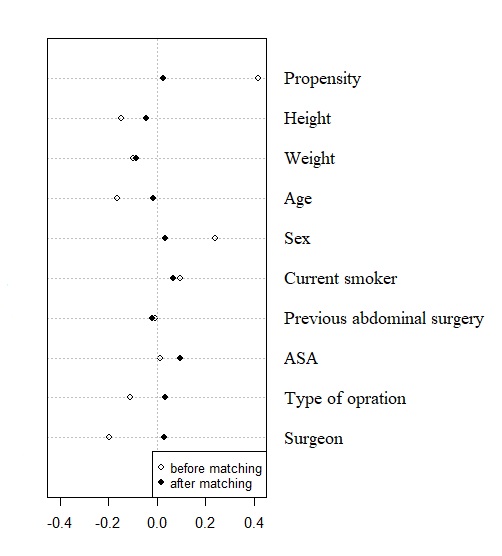

Supplement: Supplementary file 1 — Supplementary Figure S1. [file 41598_2020_62289_MOESM1_ESM.docx]
